# A DNA Vaccine Encoding SA-4-1BBL Fused to HPV-16 E7 Antigen Has Prophylactic and Therapeutic Efficacy in a Cervical Cancer Mouse Model

**DOI:** 10.3390/cancers11010096

**Published:** 2019-01-15

**Authors:** Rodolfo Garza-Morales, Jose J. Perez-Trujillo, Elvis Martinez-Jaramillo, Odila Saucedo-Cardenas, Maria J. Loera-Arias, Aracely Garcia-Garcia, Humberto Rodriguez-Rocha, Esma Yolcu, Haval Shirwan, Jorge G. Gomez-Gutierrez, Roberto Montes-de-Oca-Luna

**Affiliations:** 1 Departamento de Histología, Facultad de Medicina, Universidad Autónoma de Nuevo León, Monterrey 66460, NL, Mexico; rod.ggarza@gmail.com (R.G.-M.); josejuan2886@hotmail.com (J.J.P.-T.); elvismtzj@gmail.com (E.M.-J.); odilam@hotmail.com (O.S.-C.); loera.arias@gmail.com (M.J.L.-A.); aracely_79_20@yahoo.com (A.G.-G.); humbertordz54@hotmail.com (H.R.-R.); 2 The Hiram C. Polk Jr, MD, Department of Surgery, School of Medicine, University of Louisville, Louisville, KY 40202, USA; 3 Departamento de Genética Molecular, Centro de Investigación Biomédica del Noreste, Instituto Mexicano del Seguro Social (IMSS), Monterrey 64720, NL, Mexico; 4 Department of Microbiology and Immunology, Institute of Cellular Therapeutics, University of Louisville, Louisville, KY 40202, USA; e0yolc01@louisville.edu (E.Y.); haval.shirwan@louisville.edu (H.S.)

**Keywords:** DNA vaccine, E7, SA-4-1BBL, HPV-16, co-stimulation, IFN-γ, cancer vaccine, T cell responses

## Abstract

The SA-4-1BBL, an oligomeric novel form of the natural ligand for the 4-1BB co-stimulatory receptor of the tumor necrosis factor (TNF) superfamily, as a recombinant protein has potent pleiotropic effects on cells of innate, adaptive, and regulatory immunity with demonstrated therapeutic efficacy in several tumor models. However, the production of soluble form of SA-4-1BBL protein and quality control is time and resource intensive and face various issues pertinent to clinical development of biologics. The present study sought to take advantage of the simplicity and translatability of DNA-based vaccines for the production and delivery of SA-4-1BBL for cancer immune prevention and therapy. A chimeric HPV-16 E7 DNA vaccine (SP-SA-E7-4-1BBL) was constructed that contains the signal peptide (SP) of calreticulin (CRT), streptavidin (SA) domain of SA-4-1BBL, HPV-16 E7 double mutant gene, and the extracellular domain of mouse 4-1BBL. Immunization by gene gun with SP-SA-E7-4-1BBL induced greater prophylactic as well as therapeutic effects in C57BL/6 mice against TC-1 tumor model compared with immunization with E7wt, SP-SA-4-1BBL or reference-positive control CRT-E7wt. The therapeutic efficacy of the DNA vaccine was associated with increased frequency of E7-specific T cells producing interferon (IFN)-γ. Overall, our data suggest that this DNA-based vaccine strategy might represent a translational approach because it provides a simpler and versatile alternative to a subunit vaccine based on SA-4-1BBL and E7 proteins.

## 1. Introduction

The development of vaccines that modulate the innate, adaptive, and regulatory immune responses represents an attractive strategy for cancer immunotherapy. Co-stimulation is an integral and critical component of immune regulation that requires the correct functioning of a multitude of pathways such as B7-1/B7-2:CD28/CTLA-4, ICOS/ICOS ligand, CD27/CD70, 4-1BB/4-1BBL, among others. The correct modulation of these pathways provides an attractive approach to control T-cell activation and differentiation for the treatment of cancer [1,2].

Co-stimulatory molecules of the tumor necrosis factor (TNF) superfamily are promising cancer immunotherapy molecules, as they have a key role in the generation and expansion of primary immune responses as well as in the establishment of long-term immunological memory [3,4]. 4-1BB and 4-1BBL are an important receptor-ligand pair that belongs to TNF superfamily, where 4-1BB (CD137) is inducible on activated CD4^+^ and CD8^+^ T cells, activated dendritic cells, and activated NK and NKT cells [5,6,7]. 

The expression of 4-1BB ligand (4-1BBL, CD137L) occurs on mature dendritic cells as well as on macrophages and activated B cells [8,9]. The engagement of 4-1BBL with its receptor 4-1BB promotes the proliferation and survival of CD8^+^ T cells, prevents activation induced cell death, and induces the production of proinflammatory cytokines IL-12, IL-2, and IL-8 by dendritic cells, CD4^+^ T cells, and macrophages, respectively [10,11,12,13,14,15].

Since 4-1BBL exists as a trimeric cell surface membranous protein and exerts no function in soluble form [3], a novel form of 4-1BBL was generated by fusing the extracellular domain of murine 4-1BBL to the C-terminus of a modified form of streptavidin core (SA; SA-4-1BBL). SA-4-1BBL exists as tetramers or high order multimers that allows cross-linking to 4-1BB [16,17,18]. This chimeric soluble form of 4-1BBL activates dendritic cells and enhances antigen uptake, stimulates primary T cell responses, maintains long-term memory and activates T effector (Teff) cells to overcome the suppressive effect of CD4^+^CD25^+^FoxP3^+^ T regulatory (Treg) cells [19].

SA-4-1BBL has been tested as an immunomodulatory component of an HPV-16 E7 recombinant protein subunit-based vaccine on an HPV-induced mouse cancer model. The SA-4-1BBL E7-based subunit vaccine exhibited significant therapeutic efficacy by eradicating established tumors in approximately 70% of mice. This antitumor response was associated with robust primary and memory CD4^+^, CD8^+^ T cell and Th1 cytokine responses [19,20]. In addition, tumors were infiltrated by CD4^+^ and CD8^+^ T cells, a marked reduction of CD4^+^CD25^+^FoxP3^+^ regulatory T cells, and enhanced NK cell killing were also observed [19,20].

Similar results also were obtained when using SA-4-1BBL as the immunomodulatory component of a survivin protein-based vaccine against 3LL lung carcinoma expressing survivin. Most importantly, the therapeutic effectiveness was achieved without acute toxicity or side effects, which is in contrast with monoclonal antibodies against 4-1BB [21,22,23].

Even though SA-4-1BBL linked to biotinylated HPV-16 E7 has clearly demonstrated its effectivity in several tumor models, its production is laborious, time consuming, and expensive to manufacture because it involves the manipulation of two genes. SA-4-1BBL has to be expressed under a *Drosophila* expression system (DES) and purified using a Sepharose column. Meanwhile, E7 antigen has to be expressed and purified separately, prior to biotinylation and conjugation to SA-4-1BBL [18,20]. In contrast, the use of DNA-based vaccines presents advantages, such as the relative stability of DNA, the specificity of the produced antigen, the fact that they are generally less time consuming, and cheaper to produce on large scale when compared to synthetic peptides or recombinant proteins [24]. Recently, we described a DNA vaccine expressing HPV-16 E6 and E7 antigens fused to the signal peptide (SP) and KDEL retention signal from calreticulin (CRT). We observed that this DNA vaccine generated high levels of interferon (IFN)-γ production as well as efficient antigen-specific antitumor responses [25].

This study represents an effort to generate a DNA-based vaccine exploiting the robust immunomodulatory features of SA-4-1BBL. We show that SP-SA-E7-4-1BBL DNA vaccine displayed robust therapeutic and prophylactic effects against HPV-16 E7-expressing TC-1 tumors when compared to controls containing either E7wt or SP-SA-4-1BBL alone. The observed enhanced antitumor effect was mediated by E7-specific T cells producing IFN-γ.

## 2. Results

### 2.1. Detection of Fusion Proteins from DNA Vaccine Plasmids

**Figure 1:**
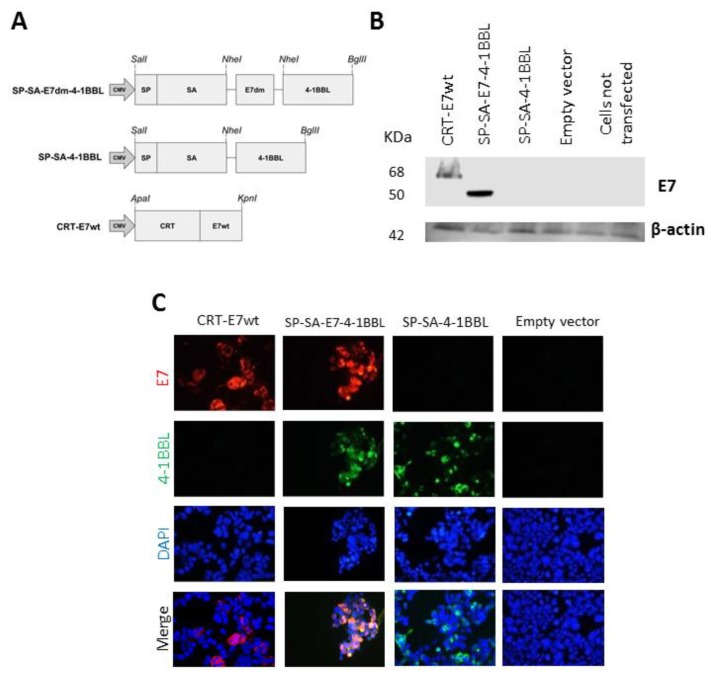
Design of DNA constructs and expression in HEK-293 cells: (**A**) Schematic of DNA constructs used in this study. This scheme shows the arrangement of streptavidin (SA), double mutant form of HPV-16 E7, and the extracellular domain of mouse 4-1BBL. A signal peptide (SP) was fused to the N-terminus of the recombinant genes. The controls were CRT-E7wt and empty plasmid. (**B**) A representative image of western blot analysis. 5 × 10^5^ HEK-293 cells were transfected with constructs expressing CRT-E7wt, SP-SA-E7-4-1BBL, SP-SA-4-1BBL, and empty vector as negative control. Twenty-four hours later, cells were lysed, and protein expression was analyzed by western blot using E7 antibody. (**C**) Immunofluorescent staining of transfected HEK-293 cells. HEK-293 cells were transfected with constructs expressing CRT-E7wt, SP-SA-E7-4-1BBL, SP-SA-4-1BBL, and empty vector as negative control. Twenty-four hours later, cell slides were fixed and incubated with E7 and 4-1BBL antibodies, and the specific secondary antibodies conjugated with fluorochromes. Evaluation of DNA construct expression was performed by fluorescence microscopy. Images were taken at 40× magnification, DAPI was used as a marker for nuclei. DAPI, 4′,6-diamidino-2-phenylindole; CMV, cytomegalovirus promoter; CRT, calreticulin; SP, signal peptide.

The SP-SA-E7-4-1BBL fusion construct was generated by fusing a non-oncogenic double mutant version of HPV-16 E7 (E7dm) to SA-4-1BBL (Figure 1A). The addition of this mutant version of E7 antigen eliminates the risk of cellular transformation [25]. The expression of SP-SA-E7-4-1BBL and SP-SA-4-1BBL was confirmed on transfected HEK-293 cells by western blot analysis and immunofluorescence. Western blot analysis revealed 50 and 68 kDa bands corresponding to the theoretical sizes of SP-SA-E7-4-1BBL and CRT-E7wt, respectively (Figure 1B). Expression of SP-SA-E7-4-1BBL, SP-SA-4-1BBL, and CRT-E7wt were further validated by an immunofluorescent staining using monoclonal antibodies against 4-1BBL and E7 (Figure 1C).

### 2.2. Vaccination with SP-SA-E7-4-1BBL Protected Mice against TC-1 Tumor Challenge

**Figure 2:**
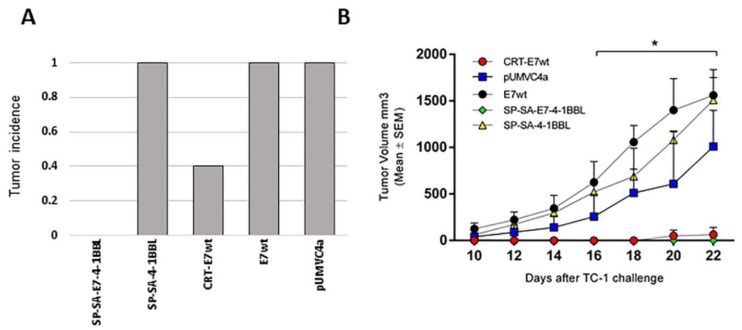
Immunization with the SP-SA-E7-4-1BBL DNA vaccine shows prophylaxis against TC-1 tumor: (**A**) Endpoint tumor incidence. Groups (n = 5) of five 5- to 6-week-old C57BL/6 mice were immunized on shaved abdominal skin with 1 µg of DNA using gene gun system, and a week later they received a booster immunization under the same conditions. One week later, mice were challenged with 5 × 10^4^ TC-1 cells in the right flank by subcutaneous injection. Tumor incidence is reported as the fraction of mice bearing tumors of diameter ≥2 mm. (**B**) Tumor volume after subcutaneous injection of TC-1 cells in C57BL/6 mice. Tumor volumes are represented by mean ± SEM. Mice were euthanized when the tumor volume was higher than 1500 mm^3^. * *p* < 0.05, SP-SA-E7-4-1BBL and CRT-E7wt compared with E7wt, SP-SA-4-1BBL and empty vector. Two-way ANOVA and *post-hoc* Tukey’s test were performed.

To test the prophylactic efficacy of SP-SA-E7-4-1BBL, groups of five 5- to 6-week-old C57BL/6 female mice were immunized by gene gun with 1 µg of the following DNA constructs: SP-SA-E7-4-1BBL, SP-SA-4-1BBL, CRT-E7wt, E7wt, or empty vector pUMVC4a. Mice were immunized twice (at days 0 and 7), challenged with TC-1 cells (at day 14), and monitored for tumor growth. Tumor protection response was evaluated by comparing tumor volumes registered among all groups throughout the prophylactic assay. Mice immunized with SP-SA-E7-4-1BBL DNA construct did not develop tumors and remained tumor-free throughout the study. In marked contrast, the other three groups of mice vaccinated with plasmids containing empty vector (pUMVC4a), E7wt, or SP-SA-4-1BBL developed tumors. Vaccination with CRT-E7wt was also effective in preventing tumor growth as only 2/5 mice developed tumors (Figure 2A). At the end of the study, mean tumor volumes from CRT-E7wt-treated mice were approximately 95% smaller compared to mice treated with empty vector, SP-SA-4-1BBL, and E7wt (*p* < 0.05) (Figure 2B). Vaccination with E7wt, SP-SA-4-1BBL and pUMVC4a DNA construct was not effective in preventing tumor growth, and there were no statistically significant differences between these groups.

### 2.3. Vaccination with SP-SA-E7-4-1BBL Induces a Therapeutic Effect against Established TC-1 Tumors

**Figure 3:**
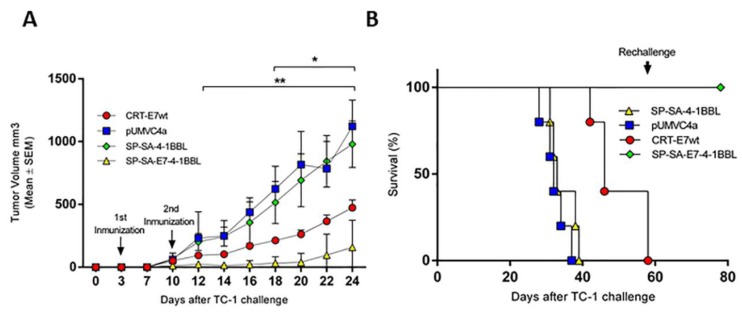
SP-SA-E7-4-1BBL DNA vaccine shows therapeutic efficacy against TC-1 tumor with long-term immunological memory: (**A**) Tumor volume after subcutaneous injection of TC-1 cells in C57BL/6 mice. At days 3 and 10 after tumor challenge, mice were immunized on shaved abdominal skin with 1 µg of DNA using gene gun system. Tumor volumes are represented by mean ± SEM. (**B**) Survival graph after TC-1 cell challenge. Mice were euthanized when the tumor volume was higher than 1500 mm^3^. * *p* < 0.05, SP-SA-E7-4-1BBL compared with CRT-E7wt, SP-SA-4-1BBL and empty vector, ** *p* < 0.05, SP-SA-E7-4-1BBL compared with SP-SA-4-1BBL and empty vector. Two-way ANOVA and *post-hoc* Tukey’s test were performed.

To evaluate whether SP-SA-E7-4-1BBL is effective in a therapeutic setting, cohorts of C57BL/6 female mice were challenged with 5 × 10^4^ TC-1 cells (day 0). Three days later, the mice were immunized by gene gun with 1 µg of the following DNA constructs: SP-SA-E7-4-1BBL, SP-SA-4-1BBL, CRT-E7wt, or empty vector. Mice received the second vaccination one week later and were monitored for tumor growth and survival. At day 12 after TC-1 challenge and throughout the study, a difference in tumor volume was observed between SP-SA-E7-4-1BBL and SP-SA-4-1BBL and empty vector groups (*p* < 0.05). By day 18 and throughout the study, greater tumor suppression was observed in mice immunized with SP-SA-E7-4-1BBL when compared to CRT-E7wt, SP-SA-4-1BBL, and empty vector groups (Figure 3A; *p* < 0.05). At the end of the study, tumor sizes were approximately 13%, 57%, and 86% smaller in mice immunized with SP-SA-4-1BBL, CRT-E7wt, and SP-SA-E7-4-1BBL than in mice treated with negative control empty vector. The final tumor volume was similar in the empty vector and SP-SA-4-1BBL treatment groups. Immunization with SP-SA-E7-4-1BBL resulted in 100% survival during a 40-day observation period, whereas vaccination with SP-SA-4-1BBL and CRT-E7wt resulted in 0% and 80% survival, respectively (Figure 3B). Mice that initially were treated with SP-SA-E7-4-1BBL and were not tumor-bearing (4/5) were re-challenged at day 60 with 5 × 10^4^ TC-1 cells injected subcutaneously in the contralateral flank. These mice did not develop tumors for a period of 20 days after the second tumor challenge. These results demonstrate that the fusion of SA-4-1BBL with E7 in a DNA-based vaccine eradicate established TC-1 tumors and establish long-term immunologic memory that prevent recurrences.

### 2.4. Immunization with SP-SA-E7-4-1BBL DNA Construct Induces E7-Specific IFN-γ Production

**Figure 4:**
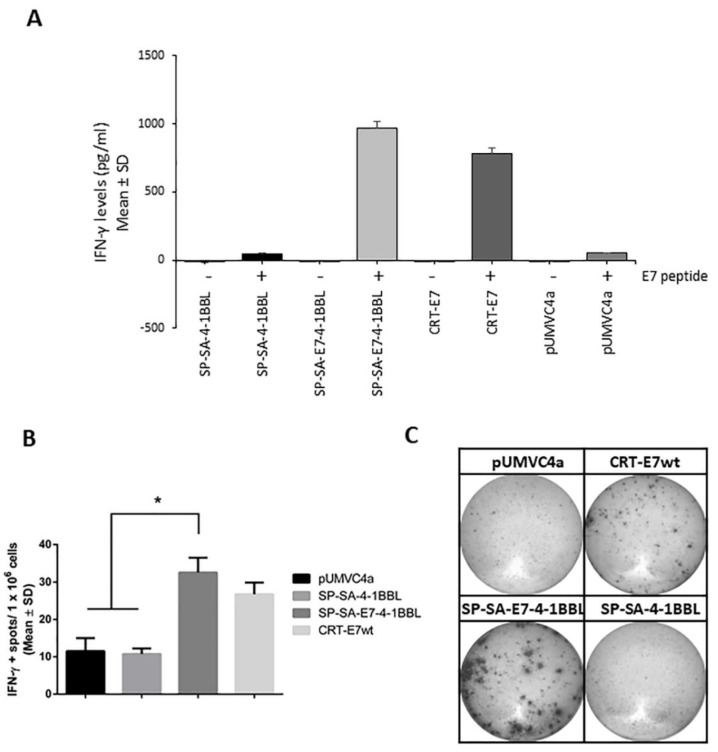
Immunization with SP-SA-E7-4-1BBL DNA vaccine generates E7-specific IFN-γ production: (**A**) C57BL/6 mice were immunized on day 0 and 7 using 1 µg of DNA on shaved abdominal skin by the gene gun system. Splenocytes were harvested 23 days after the last immunization, processed by Ficoll-Paque, and the resultant mononuclear cells were stimulated in ELISA IFN-γ assay with the E7 peptide for 48 h. (**B**) Mice were immunized as described above. Splenocytes were harvested 23 days after the last immunization and were processed as described above and stimulated in an ELISpot IFN-γ assay with the E7 peptide for 48 h. (**C**) Representative well images for each treatment group on the ELISpot IFN-γ assay. * *p* < 0.05, SP-SA-E7-4-1BBL compared with SP-SA-4-1BBL and empty vector. Two-way ANOVA and *post-hoc* Tukey’s test were performed. IFN, interferon; SD, standard deviation.

T-cell immunity is critical for an effective immune response to tumors. To test the ability of SP-SA-E7-4-1BBL DNA construct to generate anti-E7 T-cell responses in vivo, mice were immunized as described above. Twenty-three days after the last immunization, splenocytes from immunized mice were harvested and stimulated with a synthetic peptide representing the dominant CD8^+^ T cell epitope for E7 (aa 49-57). IFN-γ is an important cytokine for Th1-mediated response as well as effector function of CD8^+^ T cells that are critical to tumor eradication. ELISA assay showed a 20-fold increase in antigen-specific IFN-γ levels in mice immunized with SP-SA-E7-4-1BBL as compared with SP-SA-4-1BBL and empty vector groups (Figure 4A; *p* < 0.05). To further validate the induction of E7-specific IFN-γ production, we evaluated cell-mediated immunity by using a standard enzyme-linked immunospot (ELISpot) assay to monitor the ability of splenocytes from immunized mice to secrete IFN-γ after antigen-specific stimulation with the peptide. There was a 3-fold increase in IFN-γ positive spots in SP-SA-E7-4-1BBL-treated mice as compared to SP-SA-4-1BBL and empty vector (*p* < 0.05). Meanwhile, although SP-SA-E7-4-1BBL showed a higher number of IFN-γ positive spots, there was no significant difference when compared with reference control CRT-E7wt (*p* = 0.5648). Thus, SP-SA-E7-4-1BBL showed significantly higher levels (*p* < 0.05) of antigen-specific IFN-γ production as compared with SP-SA-4-1BBL and empty vector groups (Figure 4B,C).

## 3. Discussion

The oligomeric SA-4-1BBL co-stimulatory is a novel form of ligand for 4-1BB receptor with demonstrated robust pleiotropic effects on cells of innate, adaptive, and regulatory immunity with therapeutic efficacy in several tumor models [20,21]. However, the production of a subunit vaccine with SA-4-1BBL as the adjuvant component is laborious and may not be cost effective as compared with DNA-based vaccines. We herein designed and tested a DNA-based vaccine as an alternative to a subunit vaccine for prophylactic and therapeutic efficacy against TC-1 cervical cancer tumor model.

There are several advantageous to DNA-based vaccines. DNA vaccines are simple and relatively inexpensive to manufacture. Moreover, DNA-based vaccines are safe, stable, and well tolerated, which allows for repeated administration [24]. However, DNA vaccines have some limitations. DNA vaccines do not hold intrinsic specificity for targeting antigen presenting cells. They also do not adequately stimulate innate immune responses required to trigger strong and lasting adaptive immune responses (APCs) [26,27,28]. As a result, the potency of therapeutic DNA vaccines may be limited. Because the success of a cancer vaccine is dependent on the combinatorial use of adjuvants for better therapeutic efficacy, several strategies to enhance the potency of these vaccines have emerged in order to overcome such obstacles. In our study, we followed several approaches to enhance the immune response of our DNA vaccine, such as optimizing codon usage, dermal administration by particle-mediated delivery or “gene gun” and inclusion of SA-E7-4-1BBL fusion gene within the expression vector.

In this study, we generated a chimeric DNA construct (SP-SA-E7-4-1BBL) that consists of SA-4-1BBL and a modified form of the HPV-16 E7 oncogene. Vaccination with SP-SA-E7-4-1BBL resulted in robust prophylactic as well as therapeutic efficacy against the TC-1 tumor. The observed 100% survival in the therapeutic setting was comparable with the survival rate previously reported using the SA-4-1BBL and E7 protein subunit vaccine [20]. Furthermore, mice treated with SP-SA-E7-4-1BBL did not develop tumor when re-challenged with the TC-1 tumor cells 60 days after the initial tumor inoculation, demonstrating the existence of long-term immunologic memory. Importantly, vaccination with SP-SA-4-1BBL construct without E7 did not prevent tumor growth, demonstrating the obligatory role E7 antigen in the induced anti-tumor immune responses.

Recently, it was demonstrated that an adenoviral vaccine composed of a secreted version of 4-1BBL (Fc-4-1BBL) co-expressed with the invariant chain (Ii) adjuvant fused to glycoprotein from lymphocytic choriomeningitis virus (LCMV) exhibited tumor-specific responses. In tumor-bearing mice, this vaccine showed a pronounced reduction in tumor size when compared with the membrane bound form of 4-1BBL. Unfortunately, this effect was short lived, and when compared with a monoclonal anti-4-1BB treatment, the therapeutic efficacy levels were lower [29].

In this study, SA-E7-4-1BBL and SA-4-1BBL gene sequences are preceded by a signal peptide (SP) from calreticulin (CRT). CRT is a chaperone protein present in the endoplasmic reticulum (ER) that possesses a SP and a KDEL retention sequence. We have previously shown that ER targeting of E7 using import and retention signals enhances the antigen-specific immune responses elicited by a therapeutic HPV-16 E7 DNA vaccine [30]. Nevertheless, in this study, we only added SP so that the protein was secreted and not retained in the ER. Also, the addition of KDEL retention signal was not as important as SP, since KDEL only increased survival by 10% and did not show a significant difference in the prophylactic setting. This antitumor effect was associated with strong E7-specific CD8^+^ T-cell immune responses. Our results show that SP-SA-E7-4-1BBL elicited a similar prophylactic effect to the one observed using the CRT-E7wt DNA construct [30]. We hypothesize that this enhanced response may have been partly due to different mechanisms such as the insertion of the signal peptide sequence and the pleiotropic effects that SA-4-1BBL exerts on the immune system. In addition, we observed a correlation between the antitumor effect observed and the results from ELISpot and ELISA IFN-γ assays, which suggest that immunization with SP-SA-E7-4-1BBL DNA vaccine induced an E7-specific strong cellular immune response in comparison to immunization with SP-SA-4-1BBL and CRT-E7wt.

In conclusion, we developed a novel DNA vaccine, SP-SA-E7-4-1BBL, encoding a modified form of E7 fused to the immunomodulatory protein SA-4-1BBL. Vaccination with SP-SA-E7-4-1BBL was effective in generating a Th1 immune response defined by the expression of IFN-γ that was associated with both prophylactic and therapeutic efficacy against the TC-1 tumor. This simplified DNA-based vaccine strategy provides an efficient and versatile alternative to the SA-4-1BBL E7-based subunit protein vaccine with significant clinical potential.

## 4. Materials and Methods

### 4.1. DNA Constructs

The SP-SA-E7-4-1BBL sequence, including codon optimization and restriction sites, was designed by our laboratory team and synthesized by GenScript (Piscataway, NJ, USA). For the SP-SA-E7-4-1BBL construct, we used a non-oncogenic double mutant form of HPV-16 E7 gene (E7dm) [30] and the SA-4-1BBL gene sequence described previously [18]. The E7 double mutant gene was fused in between the streptavidin core (SA) and 4-1BBL sequence using a flexible linker based on an alanine and glutamine sequence (AEAAAKEAAAKAAA). A signal peptide (SP) from human calreticulin (CRT) was added at the N-terminus. The synthetic fusion gene was 1434 bp in length. First, SP-SA-E7-4-1BBL was subcloned into pUMVC4a vector from Aldevron (Fargo, ND, USA) using *SalI* and *BglII* restriction sites. Plasmid characterization was performed with *ScaI* restriction enzyme. The vector used in this study (pUMVC4a) was a cytomegalovirus (CMV)-regulated vector widely used for DNA immunization due to its immunostimulant activity and its efficiency for generating high numbers of plasmid copies, making it ideal for DNA gene gun cartridge preparations. Next, to obtain the SP-SA-4-1BBL construct, we deleted the E7 double mutant gene from SP-SA-E7-4-1BBL by digesting it with *NheI* restriction enzyme. The plasmid containing CRT-E7wt gene (pShuttle: rCRT-E7wt) was described previously [31] and was also subcloned into pUMVC4a using *ApaI* and *KpnI* restriction sites. The E7wt construct was obtained by digesting the CRT-E7wt construct with *BglII.* Restriction enzymes, shrimp alkaline phosphatase (rSAP) and T4 DNA ligase were purchased from New England Biolabs (Ipswich, MA, USA). Throughout the experiments, E7wt DNA construct and empty vector pUMVC4a were used as negative controls, and CRT-E7wt DNA construct was used as a reference control for positive antitumor response.

### 4.2. Cell Lines and Culture Conditions

Murine lung cancer TC-1 (Cat# CRL-2785) cells derived from primary epithelial cells of C57BL/6 mice co-transformed with HPV-16 E6/E7 and c-Ha-ras oncogenes and human embryonic kidney cell line (HEK-293) (Cat# CRL-1573) were purchased from the American Type Culture Collection (ATCC, (Manassas, VA, USA). TC-1 cells were grown in RPMI-1640 medium (Cat# 10-040-CV). HEK-293 cells were grown in Dulbecco’s Modified Eagle’s Medium (DMEM) (Cat# 10-013-CV). All media were supplemented as previously described [25]. All cells were maintained at 37 °C in a 5% CO_2_ atmosphere. All cell culture reagents were obtained from Corning Cellgro (Manassas, VA, USA).

### 4.3. Immunofluorescence and Western Blot Analysis

A total of 5 × 10^4^ and 5 × 10^5^ HEK-293 cells were seeded overnight in a 24-well plate and 6-well plate for immunofluorescence and western blot analysis. Next, cells were transfected with 1 µg of their respective DNA construct using TurboFect reagent (Thermo Scientific, Waltham, MA, USA). After 24 h, cells were processed as described below.

For western blot, cells were harvested and lysed with radioimmunoprecipitation assay (RIPA) buffer as described previously [25]. Cell lysates were centrifuged, and the protein concentration was determined using Pierce bicinchoninic acid assay (BCA) protein kit (Thermo Scientific). Equal amounts of cellular protein were electrophoresed on 10% SDS-polyacrylamide gels and transferred to hybond-polyvinylidene fluoride (PVDF) membranes (GE Healthcare Life Sciences, Pittsburgh, PA, USA). The primary antibodies used were mouse anti-E7 monoclonal antibody (NM2) (sc-65711, Santa Cruz Biotechnology, Dallas, TX, USA) and mouse anti-β-actin monoclonal antibody (A2228, Sigma-Aldrich, St. Louis, MO, USA). Next, the membranes were incubated with anti-mouse immunoglobulin (Ig) or anti-rabbit Ig, horseradish peroxidase conjugated, species-specific whole antibody (Thermo Scientific). Electrochemiluminescence (ECL) reagents were used to detect the signals according to the manufacturer’s instructions (GE Healthcare Life Sciences).

For immunofluorescence, cells were washed with ice-cold phosphate buffered saline 1X followed by fixation and permeabilization with cold methanol: acetone solution (1:1) for 1 min. After a second washing step, cells were blocked with 3% horse serum for 1 h at 4 °C and were then incubated overnight with mouse anti-E7 monoclonal antibody (NM2) (Cat# sc-65711, Santa Cruz Biotechnology) and rat anti-TNFSF9 (4-1BBL) monoclonal antibody (ab86575, Abcam, Cambridge, MA, USA) at a 1:500 dilution. The cells were washed and incubated for 2 hr with goat anti-mouse IgG (H+L) Alexa 594 (red) and goat anti-rat IgG (H+L) Alexa 488 (green) at 1:3000 dilution (Thermo Fisher Scientific). The glass coverslips were washed and mounted with Vectashield antifade mounting medium with 4′,6-diamidino-2-phenylindole (DAPI, Vector Laboratories, Burlingame, CA, USA) and analyzed using an Apotome fluorescence microscope (Carl Zeiss, Thornwood, NY, USA).

### 4.4. DNA Vaccine Preparation

All DNA constructs were purified with a midi-prep endotoxin-free kit from Qiagen (Mexico City, Mexico). To produce DNA-coated gold particles for the Helios gene gun system from Bio-Rad (Hercules, CA, USA), DNA amounts were adjusted to 1 µg DNA/cartridge according to the manufacturer’s protocol and stored in desiccant chambers at 4 °C until use. DNA vaccination was performed on shaved abdominal region using a helium-driven gene gun at a 400-psi output pressure.

### 4.5. Mice

C57BL/6 mice were purchased from Circulo ADN (Mexico City, Mexico) and housed in our barrier animal facility at the School of Medicine, Autonomous University of Nuevo Leon, under a 12-h light/12-h dark cycle with *ad libitum* access to food and water. All animal procedures were performed in accordance with institutional guidelines and the principles set forth in the National Institutes of Health Guide for the Care and Use of Laboratory Animals (NIH Publications No. 8023, revised 1978). This study was analyzed and approved by the Ethics Committee of the School of Medicine, Autonomous University of Nuevo Leon (Monterrey, NL, Mexico) (protocol no. HT17-0001).

### 4.6. In Vivo Prophylactic Vaccinations

Groups of five mice were immunized on the shaved abdominal skin with 1 µg of a DNA construct using the gene gun system, and one week later they received another immunization. One week after the last immunization, mice received 5 × 10^4^ TC-1 cells in 100 µL phosphate buffered saline 1× in the right flank by subcutaneous injection. Tumor progression was evaluated by measuring tumor diameter every 2 days using a digital caliper, and tumor volume was reported by using the following formula: tumor volume = (tumor minor diameter^2^) × (tumor major diameter)/2. Tumor incidence was reported as the fraction of mice bearing tumors of diameter ≥ 2 mm. All tumor-bearing mice were euthanized when tumors reached 1500 mm^3^ in tumor volume or earlier if ulceration was present or mice showed signs of discomfort.

### 4.7. In Vivo Therapeutic Vaccinations

Groups of five mice received 5 × 10^4^ TC-1 cells in 100 µL phosphate buffered saline 1X in the right flank by subcutaneous injection. Three days later, using the gene gun system, mice were immunized on the shaved abdominal skin with 1 µg of their respective DNA construct, and one week later they received a second immunization. Tumor progression was measured as described above. For survival analysis, all tumor-bearing mice were euthanized when tumors reached 1500 mm^3^ in tumor volume or earlier if ulceration was present or mice showed signs of discomfort.

### 4.8. Antigen-Specific Interferon (IFN)-γ Quantification by ELISpot and ELISA Assays

On days 0 and 7, using the gene gun system, groups of five mice were immunized in the shaved abdominal region with 1 µg of their respective DNA construct. Mice were sacrificed 23 days after the last immunization, and their spleens were collected and mashed on a 30-µm nylon mesh. Pooled splenocytes for each treatment were incubated overnight in RPMI medium at 4 °C to minimize cellular debris during assay. Later, mononuclear cells were obtained from the splenocytes using a Ficoll-Paque protocol (GE Healthcare Bio-Sciences, Marlborough, MA, USA). For ELISpot assay, 5 × 10^5^ splenocytes were seeded and stimulated for 48 h with 1 µg/mL E7 immunodominant epitope (RAHYNIVTF, amino acids 49–57; GenScript, Piscataway, NJ, USA) on a mouse interferon (IFN)-γ ELISpot plate (#EL485, R&D Systems, Minneapolis, MN, USA). The plate was processed according to the manufacturer’s protocol, and the wells were photographed using a micropublisher 5.0 RTV camera (QImaging, Surrey, BC, Canada) on a stereomicroscope (Leica, Buffalo Grove, IL, USA). The dots were counted manually using an ImageJ cell counter (developed by the National Institutes of Health, Bethesda, MA, USA). Non-stimulated mononuclear cells were used as negative controls for antigen-specific IFN-γ level determination.

For ELISA assay, 1 × 10^6^ splenocytes were seeded and stimulated for 48 h with 1 µg/mL E7 immunodominant epitope as previously described. Next, the supernatant was collected, aliquoted, and stored at −80 °C until further use. ELISA assay was performed using platinum IFN-γ ELISA kit (Cat# BMS606, eBioscience, San Diego, CA, USA). Each treatment group was corrected with the reference control that consisted of splenocytes cultured in the absence of E7 epitope stimulation.

### 4.9. Statistical Analysis

Two-way analysis of variance (ANOVA) and *post-hoc* Tukey’s test were performed using Prism software (GraphPad Software, Inc., La Jolla, CA, USA). *p* < 0.05 indicated a statistically significant difference. Experiments were performed at least twice independently.

## 5. Conclusions

This study demonstrated that a chimeric HPV-16 E7 DNA vaccine (SP-SA-E7-4-1BBL) has prophylactic and therapeutic efficacy in a cervical cancer mouse model. Additionally, this effect was associated with increased frequency of E7-specific T cells producing interferon (IFN)-γ. Overall, our data suggest that this DNA-based vaccine strategy might represent a translational approach since it provides a simpler and versatile alternative to a subunit vaccine based on SA-4-1BBL and E7 proteins.
